# Trends and projections of ischemic heart disease burden in the Belt and Road countries and China from 1990 to 2021: a study based on the 2021 global burden of disease

**DOI:** 10.1080/07853890.2025.2534100

**Published:** 2025-10-23

**Authors:** Pengpeng Liang, Jinhua Kang, Jiajun Liu, Hai Huang, Guiyun Li, Hongyan Wu

**Affiliations:** Shenzhen Hospital, Shanghai University of Traditional Chinese Medicine, Shenzhen, China

**Keywords:** Ischemic heart disease, the belt and road countries, global burden of disease, estimated annual percentage change, risk factor analysis, age-period-cohort, time trend

## Abstract

**Objective:**

To assess the burden of ischemic heart disease (IHD) in Belt and Road Initiative countries from 1990 to 2021 and to predict future trends.

**Methods:**

Utilizing data from GBD 2021, we analyzed incidence, mortality, and other indicators using Joinpoint analysis, NetDrift methodology, age-period-cohort models, and Bayesian Age-Period-Cohort (BAPC) predictive modeling.

**Results:**

In 2021, China bore the heaviest IHD burden with an incidence of 7.3 million cases and a mortality rate of 1.96 million. The United Arab Emirates experienced over a tenfold increase in incidence/prevalence rates, while Djibouti saw a fivefold rise in deaths/DALYs. Uzbekistan’s standardized incidence rate increased by 107.2%, and Lesotho’s mortality rate rose by 78.7%. A higher proportion of affected individuals were male or aged over 60 years; aging populations and demographic growth emerged as primary driving factors. Low/medium-low SDI regions exhibited significant potential for improvement; emerging risks included renal insufficiency and low grain diets. Projections indicate that by 2046, the number of IHD cases in China may exceed 14,410,378 individuals, highlighting a severe prevention and control challenge.

**Conclusion:**

The burden of IHD demonstrates regional disparities characterized by the interplay between traditional risk factors and emerging threats. It is recommended to establish a stratified governance framework that enhances cross-national data sharing and facilitates the transfer of digital health technologies to optimize cardiovascular disease prevention strategies.

## 1. Introduction

Ischemic heart disease (IHD) is the most prevalent form of cardiovascular disease and is considered a significant threat to sustainable development in the twenty first century [1,2]. IHD is the myocardial damage caused by the change in cardiac coronary circulation, which can result in an imbalance between coronary blood flow and myocardial demand. Research indicates that over the past few decades, IHD has emerged as the leading cause of death globally, accounting for 16% of all deaths. In 2019 alone, IHD resulted in more than 182 million disability-adjusted life years (DALYs) [3], making it the second largest source of health loss from non-communicable diseases and exerting persistent pressure on public health governance. In China, the number of deaths due to IHD reached approximately 1.874 million in 2019, constituting about 20.5% of global IHD-related fatalities. The DALYs attributed to IHD was estimated at around 18.8585 million years, which represents approximately 10.4% of total global DALY caused by this condition [4]. In the United States, direct medical costs associated with cardiovascular diseases are projected to rise from $273 billion in 2010 to $818 billion by 2030 [5]. Clinically, elderly patients with ischemic heart disease (IHD) frequently exhibit nonspecific symptoms and often present with multiple comorbidities. This lack of diagnostic specificity can result in delayed identification of severe cardiovascular events, such as heart failure or myocardial infarction. Notably, postmenopausal women experience an increased risk for developing IHD due to a lack of estrogen’s protective effects—particularly among those aged over seventy—where incidence rates begin approaching those seen in men [6,7]. As global populations age alongside rising dietary and metabolic issues coupled with obesity-related conditions, the burden imposed by IHD will continue to place substantial strain on both healthcare systems and economic structures worldwide.

The ‘Belt and Road Initiative’ is a significant proposal put forward by China in 2013. Since its inception, the initiative has played an important role in strengthening international cooperation, promoting economic development, enhancing healthcare, and facilitating cultural exchanges [8–10]. However, the people living in this region also face a heavy burden of multiple diseases such as IHD [11,12]. Developing countries along the Belt and Road route, particularly low-income nations in North Africa and the Middle East, are experiencing accelerated aging issues compounded by factors such as inadequate health education, insufficient medical systems, and high nursing costs. These challenges have led to a continuous increase in disease burden that severely restricts the implementation of prevention and treatment measures while imposing substantial economic pressure on both society and families [13,14]. It is noteworthy that beyond these aforementioned factors, some studies have identified several other contributors closely associated with the incidence of IHD, including autoimmune diseases, smoking habits, and preterm birth [15–17]. Additionally, with the acceleration of globalization and increased economic trade interactions, there has been a rising trend in metabolic diseases driven by environmental pollution, work-related stressors, and unhealthy lifestyle choices. This trend has become a significant factor contributing to the younger onset of IHD. Given the chronic progressive nature of IHD, which is characterized by irreversible pathological changes and multiple complications, the escalating disease burden calls for an urgent reevaluation of its epidemiological features. This reevaluation will facilitate cross-national medical collaboration and health data sharing to effectively address this systemic challenge.

The current research on IHD predominantly focuses on global perspectives or specific countries, geographical regions, risk factors, particular age groups, or overall trends in various cardiovascular diseases [18–20]. There is a notable lack of multidimensional investigations into the characteristics of certain diseases within an open collaborative framework among nations and projections for future trends. In fact, such an open cooperation model is more conducive to rapid response and resource allocation while accommodating diverse interests to address complex international situations and the needs of different countries [12]. As of August 2023, China has signed cooperation documents related to the Belt and Road Initiative with 152 member countries and 32 international organizations. With the continuous expansion of cooperative fields and channels under this initiative, it becomes imperative to engage in transnational and cross-regional collaborations focused on the prevention, diagnosis, treatment, and rehabilitation of IHD in light of the current severe disease landscape. Establishing multidimensional research that encompasses age, gender, risk factors, geography, and trend forecasting can not only alleviate healthcare economic pressures faced by individual countries but also enhance regional health governance through technology transfer and capacity building. This approach offers an innovative paradigm for global cardiovascular disease prevention and control. This paper utilizes data from GBD 2021 to interpret key indicators such as incidence rate, prevalence rate, mortality rate, and DALYs rate associated with IHD in Belt and Road countries as well as China from 1990 to 2021. It aims to analyze trends in IHD disease burden along with its projected developments over the next 25 years.

## 2. Materials and methods

### 2.1. Data sources

The GBD 2021 project provides a comprehensive assessment and publication of the prevalence of 371 diseases and injuries, as well as 88 risk factors across 204 countries and 21 regions worldwide. It estimates the number and rates of incidence, prevalence, mortality, years of life lost (YLLs), years lived with disability (YLDs), DALYs, and other indicators related to disease frequency and burden stratified by sex and age. This study references the list from the ‘Belt and Road’ website (https://www.yidaiyilu.gov.cn/country) to extract estimates for incidence, prevalence, mortality rates, and DALYs associated with IHD in 152 Belt and Road countries from GBD 2021 along with their corresponding 95% uncertainty intervals (UIs).

### 2.2. Data statistics

We employed the age-standardized rates (ASRs) of IHD in Belt and Road Initiative countries to estimate the annual percentage change (EAPC) in trends over time [21]. Decomposition analysis was utilized to identify explanatory factors driving changes in IHD trends based on population size, age structure, and epidemiological variations [22]. Frontier analysis was conducted to assess the relationship between IHD burden and national Sociodemographic Index (SDI), thereby enhancing our understanding of potential disparities [23]. We queried and retrieved data related to risk factors for IHD from the Global Burden of Disease database website (GHDx, http://ghdx.healthdata.org/gbd-results-tool) to investigate their impact on changes in IHD rankings among key countries. Connection regression analysis was applied to detect temporal trends in IHD burden, calculating the annual percentage change (APC) along with its 95% confidence intervals (CI) for each segment to evaluate the magnitude of these trends [24]. Net drift was employed to reflect yearly variations in epidemiological trends [25], allowing us to create a Net Drift map illustrating changes in IHD across Belt and Road Initiative countries. Subsequently, we further adopted an intrinsic estimator method using an age-period-cohort model to determine relative risk values associated with specific ages, periods or birth cohorts within epidemiological indicators [26,27]. Additionally, we projected future burdens of IHD over several decades. A Bayesian Age-Period-Cohort Analysis (BAPC) model utilizing Integrated Nested Laplace Approximation (INLA) was implemented for forecasting the disease burden of IHD over the next 25 years [28]. Furthermore, we used the Nordpred package within R software to analyze developmental trends in IHD by calculating ASR and patient cases stratified by gender [29]. The Autoregressive Integrated Moving Average (ARIMA) model within time series modeling was also employed for predicting future development trends related to IHD [30].

Because the study was based on publicly available dataset, this study was exempted by the ethics committee of the Shenzhen Hospital of Shanghai University of Traditional Chinese Medicine. Each step used to analyse the GBD database in the current study followed the guideline of cross-sectional study described in the Guidelines for Accurate and Transparent Health Estimates Reporting (GATHER) [31].

## 3. Results

### 3.1. Burden and trends of IHD in Belt and Road countries

#### 3.1.1. Incidence of IHD

**Figure 1.**
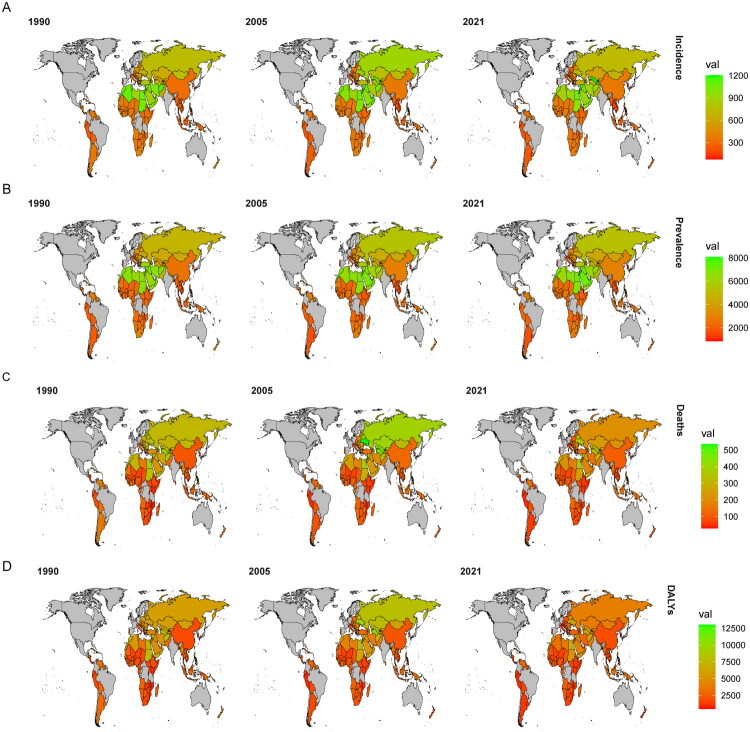
Trends in the ASR of IHD among Belt and Road countries for the years 1990, 2005, and 2021. (A) Incidence rate; (B) Prevalence rate; (C) Mortality rate; (D) DALYs rate.

Compared to 1990, the incidence of IHD has increased across all countries along the Belt and Road Initiative by 2021. Among these nations, China reported the highest number of cases, rising from 2.3016 million to 7.3046 million, representing a relative increase of 2.17 times. Following China, Russia Federation recorded 1.7194 million cases, while Pakistan had 0.8230 million cases. In terms of growth rates since 1990 within Belt and Road countries, the United Arab Emirates exhibited the most significant increase at a factor of 10.33 times; conversely, Georgia experienced the largest decline at a rate of −26.3%. Regarding standardized rates, Uzbekistan saw an increase from 581.99 per 100,000 population to 1206.01 per 100,000 population, a relative growth rate of 107.2%, marking it as having the highest increment among numerous Belt and Road nations. Portugal demonstrated a decrease in its standardized incidence rate from 178.44 per 100,000 population to 72.93 per 100,000 population, reflecting a relative decline of −59.1%, which is noted as the most substantial reduction among these countries. As of 2021, Uzbekistan, Syrian Arab Republic, and United Arab Emirates had the highest standardized incidence rates for IHD; meanwhile Brunei Darussalam, South Korea, and Portugal reported relatively lower rates (for detailed information refer to Table S1 and Figure 1A).

#### 3.1.2. Prevalence of IHD

Compared to 1990, the number of cases of IHD in China reached its highest point in 2021, increasing from 19.55 million to 63.33 million, representing a relative growth of 2.25 times. Following China, the Russian Federation reported 8.24 million cases and Pakistan reported 12.62 million cases. Among the Belt and Road Initiative countries, the United Arab Emirates exhibited the most significant increase in IHD prevalence since 1990, with an increase factor of 10.69 times; meanwhile, Niue and Georgia maintained stable rates with slight declines of 1.5% and 6.7%, respectively. Uzbekistan’s standardized prevalence rate for IHD rose from 3845.505 per 100,000 population to 5015.588 per 100,000 population, reflecting a relative increase of 30.4%, which is the largest among numerous countries involved in the Belt and Road Initiative. In contrast, New Zealand experienced a decline in its standardized prevalence rate from 3468.44 per 100,000 population to 2442.83 per 100,000 population, a relative decrease of 29.5%, marking it as having the greatest reduction among Belt and Road countries. As of December 2021, Kuwait, the United Arab Emirates, and Saudi Arabia had the highest standardized prevalence rates for IHD; conversely, Portugal, Brunei, and South Korea exhibited relatively lower rates (for detailed information see Table S1 and Figure 1B).

#### 3.1.3. Mortality of IHD

Compared to 1990, the Deaths due to IHD in China saw the most significant increase by 2021, rising from 547,800 to 1,956,900, a relative growth of 2.57 times. Following China, the Russian Federation reported an increase of 512,500 cases and Ukraine with an additional 297,500 cases. Among countries involved in the Belt and Road Initiative (BRI), Djibouti exhibited the largest increase at a rate of 5.14 times compared to its figures in 1990; conversely, Georgia experienced the greatest decline at a rate of −61.1%. In Lesotho, the age-standardized mortality rate for IHD rose from 50.87 per 100,000 population to 90.90 per 100,000 population, an increase of approximately 78.7%, marking it as having one of the highest increases among numerous BRI nations. Estonia’s standardized mortality rate decreased significantly from 368.95 per 100,000 population to just 92.06 per 100,000 population, a reduction of about −75.1%, representing the most substantial decrease within BRI countries. As of late-2021 data indicates that Nauru, Ukraine, and Syria had some of the highest standardized mortality rates due to IHD while Italy, Portugal and South Korea reported relatively lower rates (for detailed information see Table S1 and Figure 1C).

#### 3.1.4. DALYs of IHD

Compared to 1990, the DALYs due to IHD in China increased significantly from 13.6241 million in 1990 to 35.6726 million in 2021, representing a relative growth of 1.62 times. The Russian Federation and Indonesia followed with DALY counts of 9.6317 million and 7.3475 million, respectively. Among the Belt and Road Initiative countries, Djibouti exhibited the largest increase at a rate of 4.89 times compared to its figures in 1990, while Estonia experienced the most significant decrease at a rate of −65.7%. In terms of standardized rates, Lesotho saw an increase from 1003.15 per 100,000 population to 1949.43 per 100,000 population marking a relative growth of 94.3%, which is the highest among numerous Belt and Road countries. Conversely, Indonesia’s standardized rate declined sharply from 6565.71 per 100,000 population to 1547.87 per 100,000 population reflecting a relative decrease of −76.4%, which is also the most substantial decline among Belt and Road nations as of the end of 2021. As for standardized DALY rates by country as of this date, Nauru, Vanuatu, and Egypt reported the highest rates; Italy, Portugal, and South Korea had relatively lower rates (for detailed information see Table S1 and Figure 1D).

### 3.2. Analysis of IHD disease trends in countries along the Belt and Road Initiative

**Figure 2.**
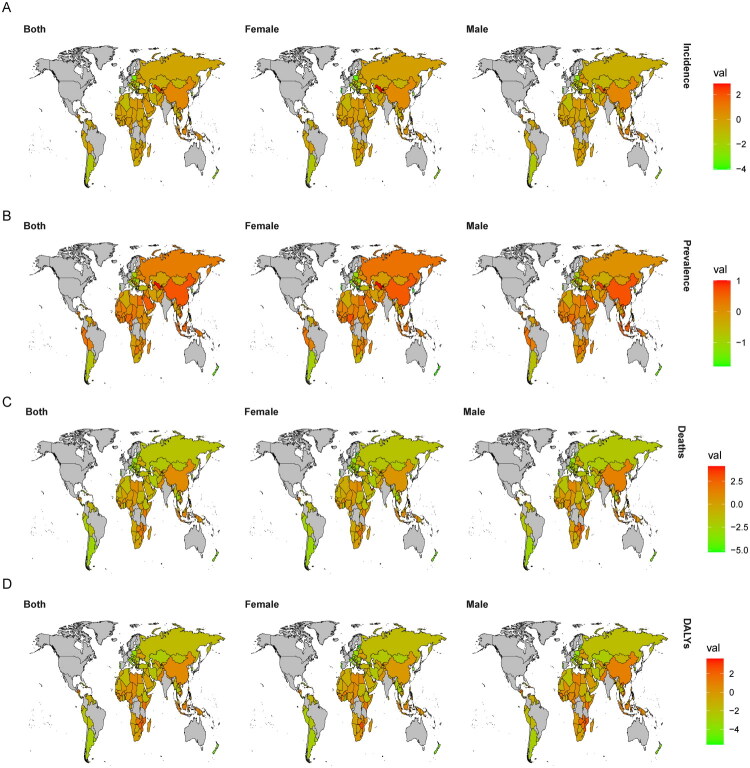
Trends in IHD EAPC across Belt and Road countries. (A) Incidence rate; (B) Prevalence rate; (C) Mortality rate; (D) DALYs rate.

From an overall perspective, 67% of the countries involved in the ‘Belt and Road’ initiative have shown a decline in incidence rates, while 42% have reported a decrease in prevalence rates. Additionally, 69% of these countries exhibit a reduction in mortality rates, and 73% show a downward trend in DALYs. However, approximately 30% of countries, including China, demonstrate an upward trend across all indicators. At the national level, China, Indonesia, the Dominican Republic, Vietnam, Timor-Leste, Lesotho, Guinea, Djibouti, Cape Verde, and Cameroon report the highest EAPC value for incidence rates, prevalence rates, mortality rates as well as DALYs. Conversely, countries such as Bulgaria, Croatia, Argentina, Italy, the Czech Republic, Romania, South Korea, New Zealand, Poland, and Portugal exhibit the lowest EAPC values for these same health metrics (for detailed information see Table S1 and Figure 2).

### 3.3. Changes in China’s ranking and age accumulation chart of IHD

**Figure 3.**
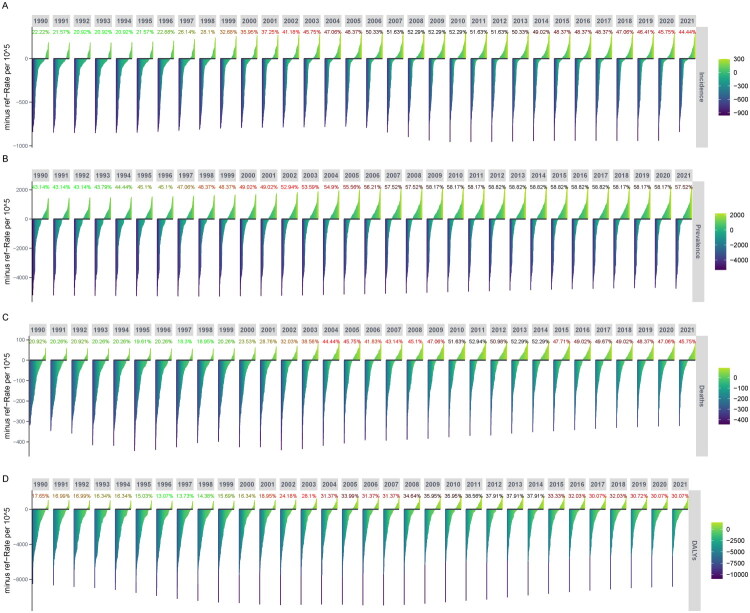
Changes in China’s IHD ranking among belt and road Initiative countries. (A) Incidence rate; (B) Prevalence rate; (C) Mortality rate; (D) DALYs rate.

**Figure 4.**
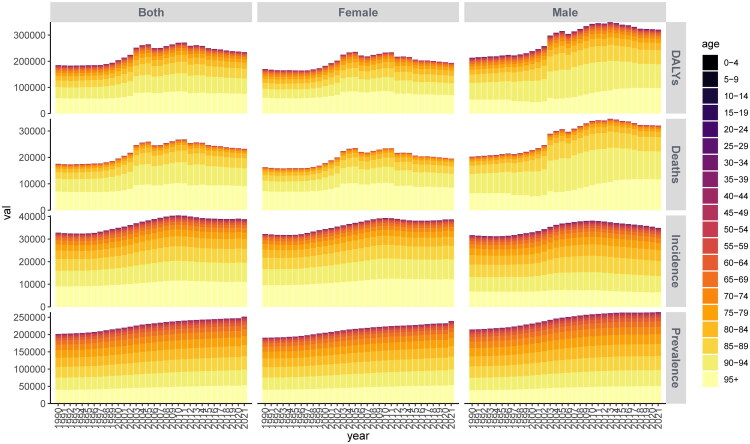
Stacked bar chart of the standardized rates for patients with IHD in China.

**Figure 5.**
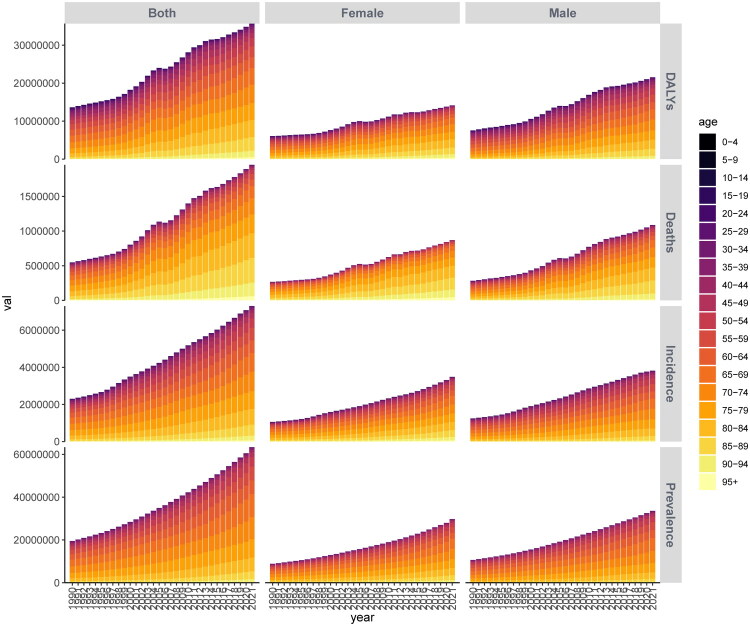
Stacked bar chart illustrating the number of patients with IHD in China.

We analyzed the changes in China’s ranking regarding IHD among Belt and Road Initiative countries from 1990 to 2019 (Figure 3). Our findings indicate that prior to 2010, the burden of IHD incidence in China consistently increased, leading to a decline in its ranking. Subsequently, a slight improvement was observed. Despite this, the prevalence of IHD in China remained severe after 2012, showing no significant change. In terms of mortality and DALYs, China’s ranking has been on a downward trend since 1990, reaching its lowest point between 2008 and 2014 when the burden was particularly high; however, there has been a modest increase in ranking over the subsequent seven years. Further analysis of age distribution for IHD cases in China reveals (Figures 4 and 5) an upward trend over the years concerning incidence rates, prevalence rates, mortality rates, and DALYs. Notably, the number of male patients significantly exceeds that of female patients each year. The age groups with relatively higher patient numbers include those aged 60–64 years, 65–69 years, 70–74 years, 75–79 years, and 80–84 years. In terms of standardized rates for IHD incidence rate, prevalence rate, mortality rate, and DALYs over time show an initial increase followed by a decrease that tends toward stabilization. The annual accumulation figures for male patients are substantially higher than those for female patients across all age groups; specifically within the age brackets of 85-89 years old, as well as those aged above or equal to ninety (90+), which constitute a major portion among all age categories.

### 3.4. Frontier analysis of IHD in Belt and Road countries

**Figure 6.**
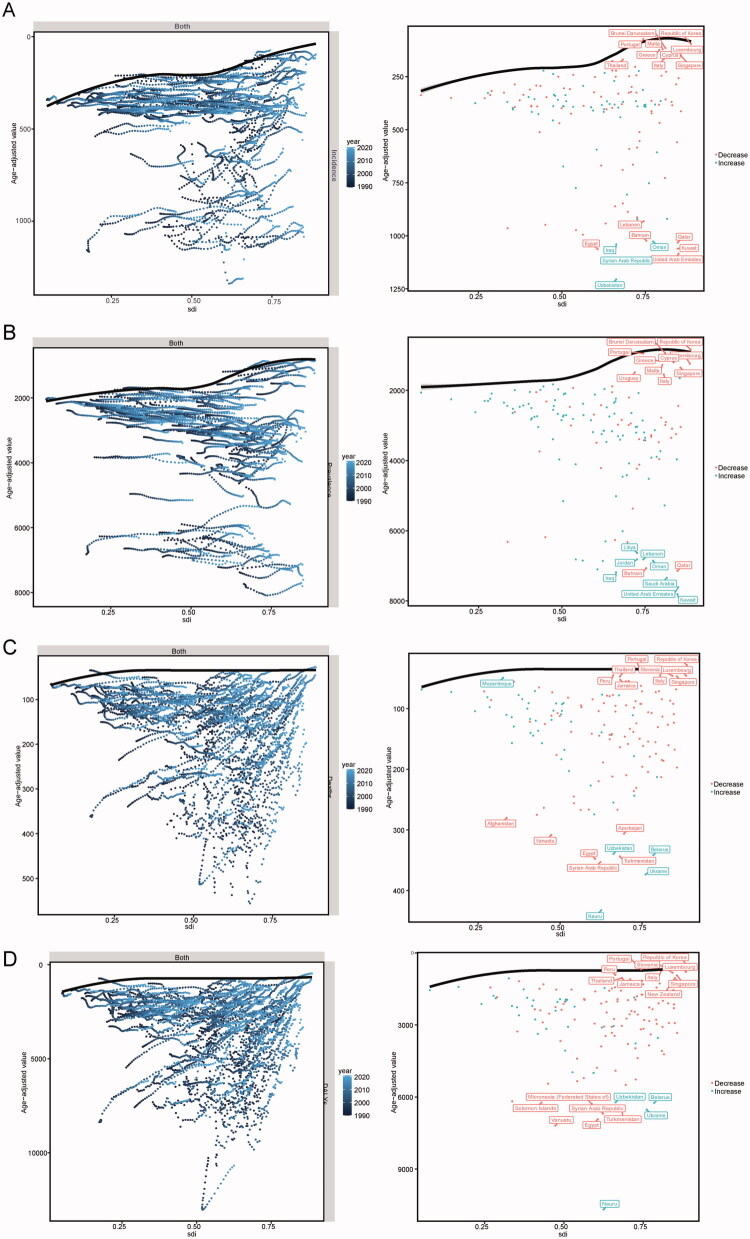
Frontier analysis of belt and road countries from 1990 to 2021, based on SDI and IHD data for the year 2021. (A) Incidence rate (B) Prevalence rate (C) Mortality rate (D) DALYs rate.

In this section, we analyze the age-standardized incidence rates (ASIR), age-standardized mortality rates (ASMR), age-standardized prevalence rates (ASPR), and age-standardized disability-adjusted life years (ASDR) for IHD in Belt and Road countries from 1990 to 2021, along with the Sustainable Development Index (SDI) data for 2021. This analysis aims to enhance our understanding of the potential improvements within these countries (see Figure 6 and Table S2). The boundary line represents the countries and regions with the lowest disease burden at a specific SDI level. The actual differences are indicated by the distance from this boundary line, reflecting the gap between a country’s current burden and its achievable minimum burden based on its development level. The solid black line denotes this boundary, while dots represent individual countries or regions; blue dots indicate an upward trend, whereas red dots signify a downward trend. Overall, as demographic factors evolved by 2021, effective disparities among nations have increased to some extent. In terms of ASIR, the ten countries or regions exhibiting the greatest effective disparity from the frontier include Uzbekistan, UAE, Kuwait, Qatar, Oman, Syria, Bahrain, Iraq, Egypt, and Lebanon. Regarding ASPR values that differ most significantly from frontier levels are Kuwait, UAE, Saudi Arabia, Qatar, Bahrain, Iraq, Oman, Lebanon, Jordania, and Libya. In relation to ASMR metrics showing substantial divergence from frontier values,the top ten affected nations or regions comprise Nauru, Ukrain, Syria, Egypt, Tajikistan, Belarus, Uzbekistan, Vanuatu, Azerbaijan, and Afghanistan. As for ASDR figures indicating significant variation relative to frontiers, Nauru, Vanuatu, Egypt, Syria, Ukrain, Tajikistan, Federated States of Micronesia, Belarus, Solomon Islands, and Uzbekistan rank among those with notable discrepancies. These findings suggest that countries or regions with lower-middle SDI exhibit greater potential for improving their disease burdens.

### 3.5. Decomposition analysis of IHD burden in important countries in the belt and road organization

**Figure 7.**
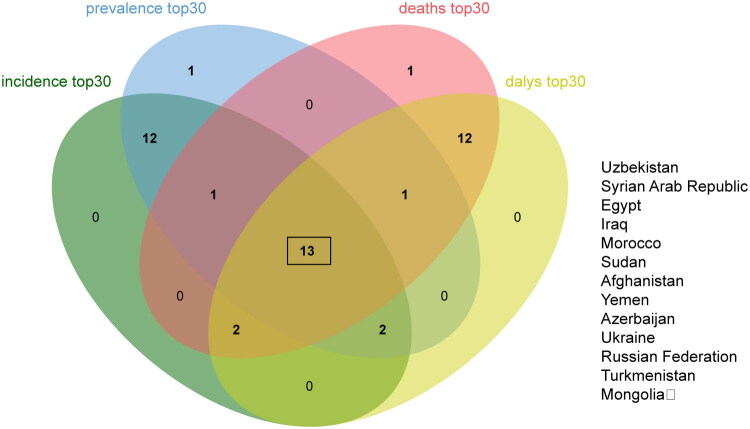
Venn diagram illustrating the prominent countries in the epidemiological indicators of IHD.

**Figure 8.**
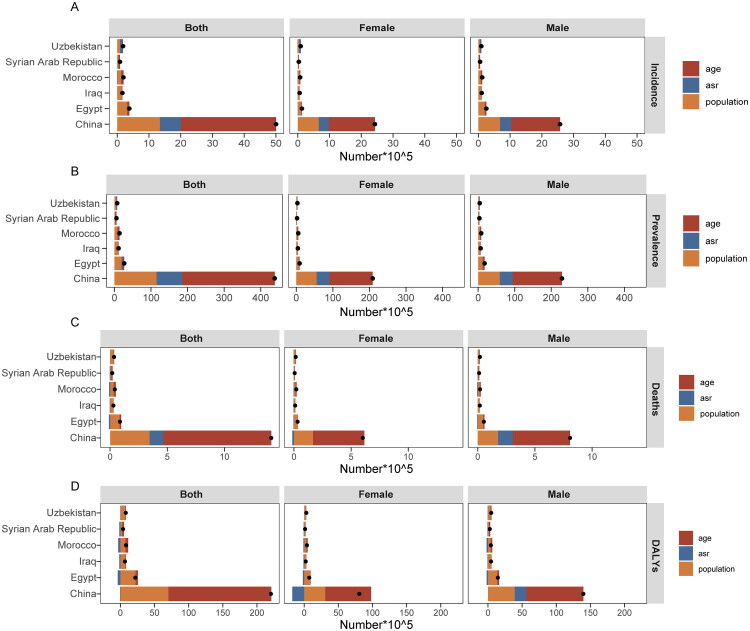
Decomposition analysis bar chart for China and five other countries. (A) Incidence rate; (B) Prevalence rate; (C) Mortality rate; (D) DALYs rate.

We conducted an intersection analysis on the top 30 countries with standardized rates of IHD epidemiology among Belt and Road Initiative nations, identifying 13 countries that consistently exhibited significant indicators across various metrics. We selected China and the five most prominent countries from this group, Uzbekistan, Syria, Egypt, Iraq, and Morocco, for a decomposition analysis based on age, epidemiological changes, and demographic factors (Figures 7 and 8). As illustrated in Figure 8, the growth observed in these countries is primarily driven by population growth and age structure. However, epidemiological changes also play a role to some extent. The incidence rates (59.93% for China and 54.57% for Syria), prevalence rates (57.75% for China and 54.92% for Syria), mortality figures (66.96% for China and 59.77% for Syria), as well as DALYs (68.3% for China and 70.18% for Syria) are predominantly mediated by aging factors in both China and Syria. In addition, population growth also significantly contributes to the incidence rates of IHD patients in Egypt (82.91%), Iraq (90.51%), Morocco (64.41%), and Uzbekistan (50.19%). This trend is similarly reflected in their prevalence rates in Egypt at 73.31%, Iraq at 86.78%, Morocco at 60.76%, and Uzbekistan at 65.05%. Furthermore, mortality rates show contributions of over one hundred percent: Egypt at101 .21%, Iraq at111 .79%, Morocco at 88 .62%,and Uzbekistan at87 .66%. The contribution of population growth to DALYs mirrors these findings with identical percentages. Epidemiological changes contribute relatively little overall to these nations’ statistics regarding IHD mortality rate sand DALYs, with some instances reflecting negative values. Additionally, the decomposition results indicate that three primary factors exhibit marked differences in their impact on male versus female populations within these countries,with effects being more pronounced among male patients than females.

### 3.6. Risk factor analysis

**Figure 9.**
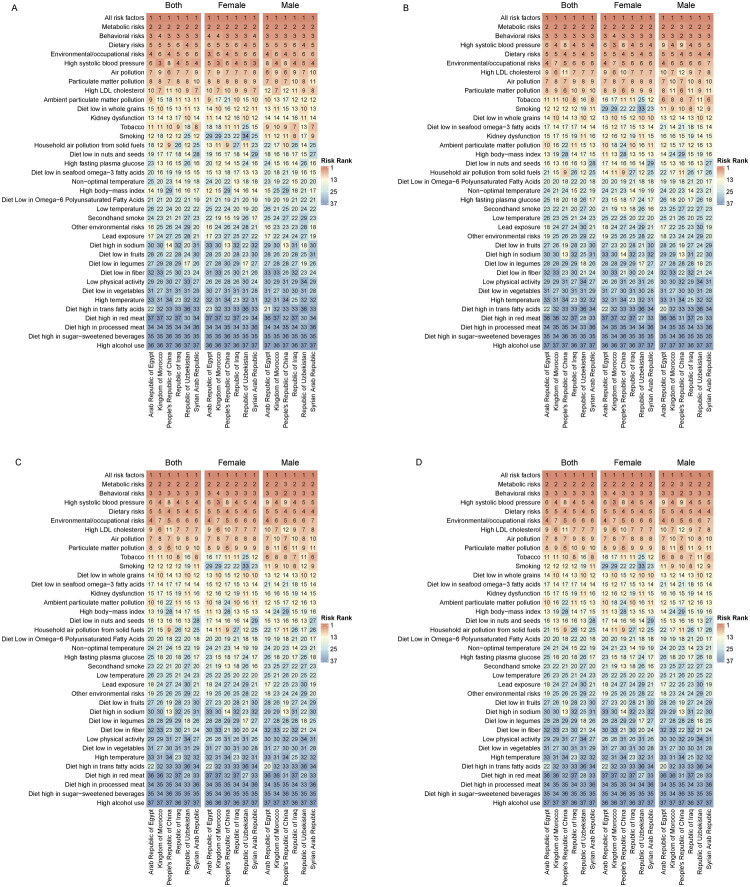
Heatmap analysis of risk factors for mortality in China and five other key countries. (A) Heatmap of mortality risk factors in 1990. (B) Heatmap of mortality risk factors in 2021. (C) Heatmap of DALYs risk factors in 1990. (D) Heatmap of DALYs risk factors in 2021 compared to 1990.

From 1990 to 2021, the risk factors contributing to mortality and disability among patients with IHD in these key countries have been significantly influenced by metabolic, behavioral, and dietary factors, which consistently ranked among the top three (excluding ‘all factors’). In China specifically, the ranking of dietary and environmental occupational risk factors has changed over this period, reflecting more regional characteristics. Additionally, air pollution and particulate matter pollution were identified as the fifth leading risk factor in 2021, indicating a persistent impact on the health of IHD patients. In Egypt, Uzbekistan, and Syria, high systolic blood pressure along with dietary risks and occupational/environmental risks ranked prominently. However, there was no significant change in the ranking of these top five risk factors over time. Behavioral risks and high systolic blood pressure have been crucial determinants affecting changes in mortality and disability among IHD patients in Morocco over nearly three decades; furthermore, high cholesterol dietary habits emerged as an additional concern in 2021. The fluctuations observed in dietary risks and environmental occupational risks are important contributors to changes seen among IHD patients in Iraq. From a gender perspective, women appear to be more susceptible to risks associated with blood pressure and environmental occupations while men show greater sensitivity to particulate matter pollution and atmospheric pollutants. Over the past thirty years, the bottom five risk factors leading to mortality and disability among IHD patients across these countries primarily include air pollution, particulate matter pollution, high-cholesterol diets, environmental particulate matter exposure, and tobacco use. It is worth noting that the data from these countries in 2021 show that kidney dysfunction and low grain diets have entered the list of significant risk factors. Moving forward it is imperative for governmental agencies as well as healthcare professionals not only to focus on individual dietary behaviors but also metabolic risks. They should equally prioritize considerations regarding air quality environment renal function impairments along with nutritional aspects related to IHD (Figure 9).

### 3.7. Joinpoint analysis

**Figure 10.**
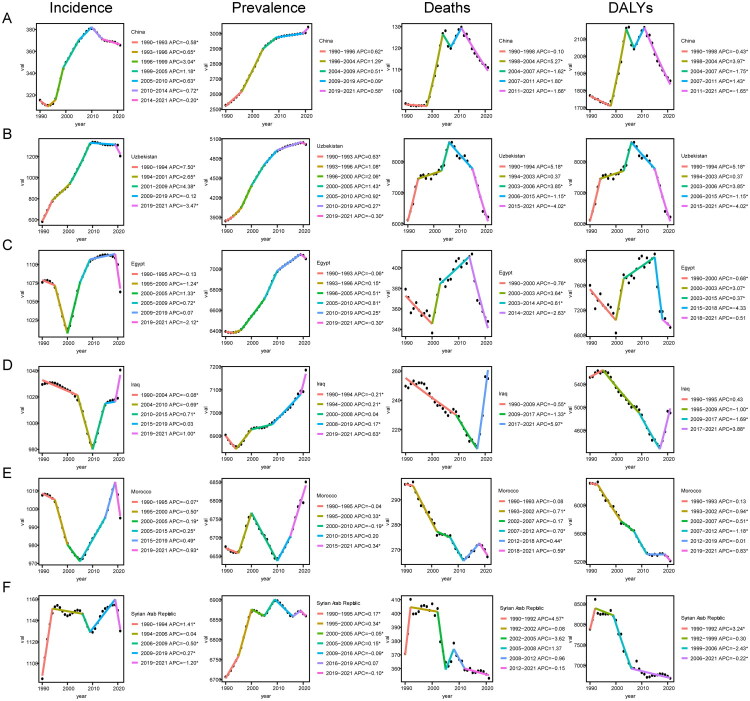
Trend analysis of IHD diseases in China and five other countries using joinpoint regression. (A) Joinpoint regression analysis for China; (B) Joinpoint regression analysis for Uzbekistan; (C) Joinpoint regression analysis for Egypt; (D) Joinpoint regression analysis for Iraq; (E) Joinpoint regression analysis for Morocco; (F) Joinpoint regression analysis for syria.

The joinpoint regression analysis for China and five other countries is illustrated in Figure 10. From 1990 to 2021, the age-standardized incidence rates of IHD in China and Uzbekistan exhibited a turning point around 2010, demonstrating an overall trend of initially increasing followed by decreasing rates. In contrast, Egypt, Iraq, Morocco, and Syria experienced turning points during the period from 2000 to 2010, showing a pattern of first declining and then rising incidence rates. Notably, after 2019, Iraq’s age-standardized incidence rate for IHD continued to show an upward trend. Over the past three decades, the age-standardized prevalence rates of IHD have generally increased in China, Uzbekistan, Egypt, Iraq, and Morocco. Although Syria has shown multiple turning points over various periods, its overall prevalence rate appears to be stabilizing. Regarding ASMR for IHD between 2005 and 2015, there were notable turning points observed in China, Uzbekistan, Egypt, Morocco, and Syria where the overall trend indicated a decline. However, the ASMR in Iraq began to rise after 2017. In terms of ASDR, significant turning points were identified among China, Uzbekistan, Egypt, Morocco, and Syria during the period from 2010 to 2020 with an overall downward trend for IHD. Conversely, the trends related to IHD diseases in Iraq showed an increase following the year of 2017 (Figure 10).

### 3.8. Epidemiological net Drift and age-period-cohort analysis

**Figure 11.**
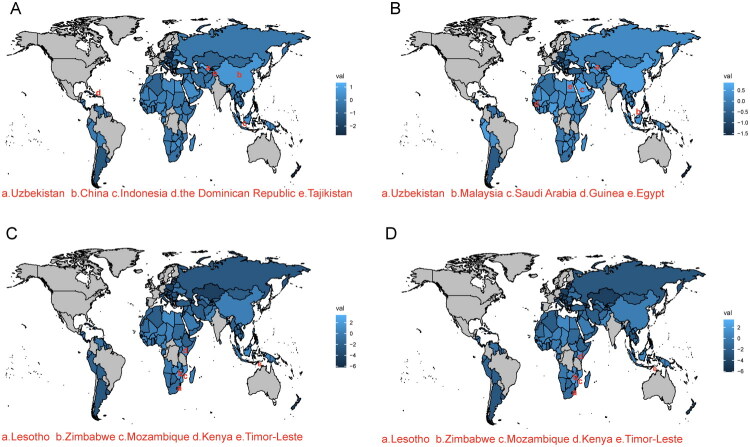
World map illustrating the net Drift analysis of Belt and Road countries. (A) ASIR; (B) ASPR; (C) ASMR; (D) ASDR. The net Drift captures components of trends attributable to temporal factors and birth cohorts.

We further analyzed the net drift world map of standardized rates of IHD in Belt and Road countries (Figure 11). We found that in terms of ASIR, Uzbekistan had a high value of 1.326 (1.126, 1.526), followed by China at 0.572 (0.457, 0.687), Indonesia at 0.566 (0.508, 0.625), the Dominican Republic at 0.558 (0.491, 0.625), and Tajikistan at 0.519 (0.440, 0.598). Regarding ASPR, Uzbekistan had a high value of 0.856 (0.811, 0.901), followed by Malaysia at 0.779 (0.717, 0.840), Saudi Arabia at 0.702 (0.669, 0.735), Guinea at 0.664 (0.577, 0.752), and Egypt at 0.565 (0.533, 0.596). In terms of ASMR, Lesotho had a high drift value of 3.413 (3.071, 3.756), followed by Zimbabwe at 2.166 (1.923, 2.411), Mozambique at 2.101 (1.967, 2.235), Kenya at 1.409 (1.255, 1.564), and Timor-Leste at 1.113 (0.866, 1.360). Additionally, in terms of ASDR, Lesotho at 3.441 (3.261, 3.622), Zimbabwe at 2.240 (2.014, 2.465), Mozambique at 2.069 (1.967, 2.171), Kenya at 1.426 (1.291, 1.560), and Timor-Leste at 1.072 (0.963, 1.181) ranked highly. Furthermore, we used the Age-Period-Cohort (APC) model to further estimate the effects of age, period, and birth cohort on the trends of IHD in China.

**Figure 12.**
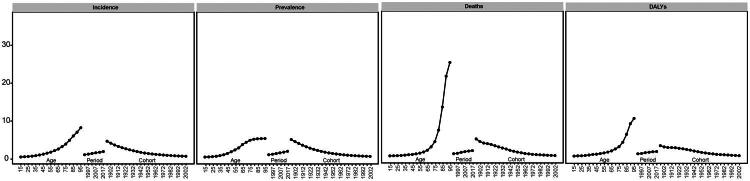
Age-period-cohort analysis of IHD in China.

The APC model analysis shows that IHD is significantly influenced by age, period, and birth cohort effects. In terms of age effects, the incidence, prevalence, and mortality rates of IHD generally show an upward trend with increasing age. Specifically, the risks for incidence, prevalence, mortality, and DALYs are lowest for the 15–19 age group, with risk values of 0.030 (0.030–0.031), 0.019 (0.019–0.019), 0.056 (0.056–0.057), and 0.10 (0.10–0.10), respectively. Conversely, individuals aged 95 and above have the highest risks for incidence, prevalence, mortality, and DALYs, with risk values of 7.768 (7.691–7.768), 4.904 (4.904–4.904), 24.779 (24.533–24.779), and 10.074 (10.074–10.074), respectively, according to the model fitting results. In the period effect coefficients, from 1990 to 2021, the risks of incidence, prevalence, mortality, and DALYs for elderly individuals with IHD show an increasing trend over time, with the maximum and minimum coefficients appearing in the sixth period group (2017–2021) and the first period group (1992–1996). The risks for the former are 2.36 times, 2.46 times, 2.29 times, and 1.84 times those of the latter for incidence, prevalence, mortality, and DALYs, respectively. In the cohort effect coefficients, the risks of incidence, prevalence, mortality, and DALYs for IHD patients generally show a downward trend as the birth cohort progresses, with the maximum and minimum coefficients appearing in the first birth cohort (1897–1901) and the last cohort (2002-2006). The risks for the former are 18.90 times, 24.05 times, 34.82 times, and 14.15 times those of the latter, respectively (Figure 12 and Table 1).

**Table 1.** Relative risk (RR) analysis of age–period–cohort in China.

Factor	Incidence		Prevalence		Deaths		DALYs	
	RR(95CI)	*P*	RR(95CI)	*P*	RR(95CI)	*P*	*RR(95CI)*	*P*
Age(years)								
15–19	0.03(0.03,0.031)	<0.001	0.019(0.019,0.019)	<0.001	0.056(0.056,0.057)	<0.001	0.1(0.1,0.1)	<0.001
20–24	0.093(0.093,0.093)	<0.001	0.046(0.046,0.046)	<0.001	0.085(0.085,0.086)	<0.001	0.15(0.15,0.15)	<0.001
25–29	0.15(0.148,0.15)	<0.001	0.113(0.113,0.113)	<0.001	0.106(0.105,0.106)	<0.001	0.181(0.181,0.181)	<0.001
30–34	0.249(0.249,0.249)	<0.001	0.219(0.219,0.219)	<0.001	0.159(0.159,0.16)	<0.001	0.259(0.259,0.259)	<0.001
35–39	0.375(0.375,0.375)	<0.001	0.383(0.383,0.383)	<0.001	0.242(0.242,0.244)	<0.001	0.375(0.375,0.379)	<0.001
40–44	0.56(0.56,0.56)	<0.001	0.619(0.619,0.619)	<0.001	0.364(0.364,0.364)	<0.001	0.538(0.538,0.538)	<0.001
45–49	0.779(0.779,0.779)	<0.001	0.951(0.951,0.951)	<0.001	0.432(0.432,0.432)	<0.001	0.6(0.6,0.6)	<0.001
50–54	1.041(1.041,1.041)	<0.001	1.391(1.391,1.391)	<0.001	0.583(0.583,0.589)	<0.001	0.756(0.756,0.756)	<0.001
55–59	1.31(1.31,1.31)	<0.001	1.896(1.896,1.896)	<0.001	0.787(0.779,0.787)	<0.001	0.932(0.932,0.932)	<0.001
60–64	1.699(1.699,1.699)	<0.001	2.435(2.435,2.435)	<0.001	1.073(1.073,1.073)	<0.001	1.15(1.15,1.162)	<0.001
65–69	2.117(2.117,2.138)	<0.001	3.287(3.287,3.32)	<0.001	1.553(1.537,1.553)	<0.001	1.462(1.462,1.462)	<0.001
70–74	2.746(2.746,2.746)	<0.001	3.935(3.896,3.935)	<0.001	2.435(2.435,2.435)	<0.001	1.974(1.974,1.974)	<0.001
75–79	3.456(3.456,3.456)	<0.001	4.437(4.437,4.437)	<0.001	3.819(3.819,3.819)	<0.001	2.56(2.56,2.56)	<0.001
80–84	4.437(4.437,4.437)	<0.001	4.711(4.711,4.711)	<0.001	6.89(6.821,6.89)	<0.001	3.743(3.743,3.743)	<0.001
85–89	5.585(5.585,5.585)	<0.001	4.855(4.807,4.855)	<0.001	12.936(12.936,12.936)	<0.001	5.812(5.812,5.871)	<0.001
90–94	6.554(6.554,6.554)	<0.001	4.855(4.855,4.855)	<0.001	21.115(21.115,21.115)	<0.001	8.585(8.585,8.671)	<0.001
95 plus	7.768(7.691,7.768)	<0.001	4.904(4.904,4.904)	<0.001	24.779(24.533,24.779)	<0.001	10.074(10.074,10.074)	<0.001
Period								
1992	0.619(0.619,0.619)	<0.001	0.625(0.619,0.625)	<0.001	0.625(0.625,0.625)	<0.001	0.705(0.705,0.705)	<0.001
1997	0.787(0.787,0.787)	<0.001	0.763(0.763,0.763)	<0.001	0.733(0.733,0.733)	<0.001	0.795(0.787,0.795)	<0.001
2002	0.961(0.961,0.961)	<0.001	0.942(0.942,0.942)	<0.001	0.99(0.99,0.99)	<0.001	1(1,1)	<0.001
2007	1.139(1.139,1.139)	<0.001	1.116(1.116,1.116)	<0.001	1.162(1.162,1.162)	<0.001	1.116(1.116,1.116)	<0.001
2012	1.284(1.284,1.284)	<0.001	1.31(1.31,1.31)	<0.001	1.323(1.323,1.323)	<0.001	1.234(1.234,1.234)	<0.001
2017	1.462(1.462,1.462)	<0.001	1.537(1.537,1.537)	<0.001	1.433(1.433,1.433)	<0.001	1.297(1.297,1.297)	<0.001
Birth cohort								
1897–1901	4.179(4.137,4.221)	<0.001	4.618(4.618,4.665)	<0.001	4.527(4.482,4.572)	<0.001	2.858(2.829,2.858)	<0.001
1902–1906	3.669(3.633,3.669)	<0.001	4.096(4.096,4.096)	<0.001	3.857(3.819,3.857)	<0.001	2.509(2.509,2.535)	<0.001
1907–1911	3.19(3.19,3.19)	<0.001	3.597(3.597,3.597)	<0.001	3.456(3.421,3.456)	<0.001	2.363(2.363,2.363)	<0.001
1912–1916	2.801(2.801,2.829)	<0.001	3.127(3.127,3.158)	<0.001	3.287(3.254,3.287)	<0.001	2.34(2.34,2.363)	<0.001
1917–1921	2.484(2.484,2.484)	<0.001	2.718(2.718,2.718)	<0.001	3.065(3.065,3.065)	<0.001	2.293(2.293,2.293)	<0.001
1922–1926	2.181(2.181,2.203)	<0.001	2.363(2.363,2.363)	<0.001	2.718(2.718,2.746)	<0.001	2.16(2.16,2.16)	<0.001
1927–1931	1.916(1.916,1.916)	<0.001	2.034(2.034,2.034)	<0.001	2.411(2.411,2.435)	<0.001	2.014(2.014,2.014)	<0.001
1932–1936	1.682(1.665,1.682)	<0.001	1.768(1.768,1.768)	<0.001	2.138(2.117,2.138)	<0.001	1.84(1.84,1.84)	<0.001
1937–1941	1.448(1.448,1.462)	<0.001	1.507(1.507,1.522)	<0.001	1.859(1.859,1.878)	<0.001	1.682(1.665,1.682)	<0.001
1942–1946	1.259(1.246,1.259)	<0.001	1.297(1.297,1.297)	<0.001	1.537(1.522,1.537)	<0.001	1.433(1.433,1.433)	<0.001
1947–1951	1.073(1.073,1.073)	<0.001	1.116(1.105,1.116)	<0.001	1.284(1.271,1.284)	<0.001	1.259(1.259,1.259)	<0.001
1952–1956	0.932(0.932,0.932)	<0.001	0.951(0.951,0.951)	<0.001	1.051(1.041,1.051)	<0.001	1.073(1.073,1.073)	<0.001
1957–1961	0.827(0.827,0.827)	<0.001	0.819(0.819,0.819)	<0.001	0.869(0.869,0.869)	<0.001	0.923(0.923,0.923)	<0.001
1962–1966	0.726(0.726,0.726)	<0.001	0.705(0.698,0.705)	<0.001	0.691(0.691,0.691)	<0.001	0.779(0.779,0.779)	<0.001
1967–1971	0.631(0.631,0.631)	<0.001	0.6(0.595,0.6)	<0.001	0.583(0.583,0.583)	<0.001	0.677(0.677,0.684)	<0.001
1972–1976	0.549(0.549,0.549)	<0.001	0.507(0.507,0.507)	<0.001	0.458(0.454,0.458)	<0.001	0.56(0.56,0.56)	<0.001
1977–1981	0.472(0.472,0.472)	<0.001	0.432(0.432,0.432)	<0.001	0.387(0.387,0.391)	<0.001	0.492(0.492,0.492)	<0.001
1982–1986	0.403(0.403,0.407)	<0.001	0.364(0.364,0.368)	<0.001	0.336(0.333,0.34)	<0.001	0.449(0.449,0.449)	<0.001
1987–1991	0.346(0.343,0.346)	<0.001	0.31(0.31,0.31)	<0.001	0.281(0.278,0.284)	<0.001	0.391(0.391,0.391)	<0.001
1992–1996	0.295(0.292,0.295)	<0.001	0.262(0.262,0.264)	<0.001	0.221(0.219,0.223)	<0.001	0.32(0.32,0.32)	<0.001
1997–2001	0.254(0.254,0.257)	<0.001	0.225(0.225,0.228)	<0.001	0.183(0.181,0.186)	<0.001	0.275(0.275,0.275)	<0.001
2002–2006	0.221(0.217,0.223)	<0.001	0.192(0.19,0.194)	<0.001	0.13(0.126,0.134)	<0.001	0.202(0.202,0.204)	<0.001

### 3.9. Epidemiological predictive model analysis of IHD patients in China

#### 3.9.1. BAPC analysis

**Figure 13.**
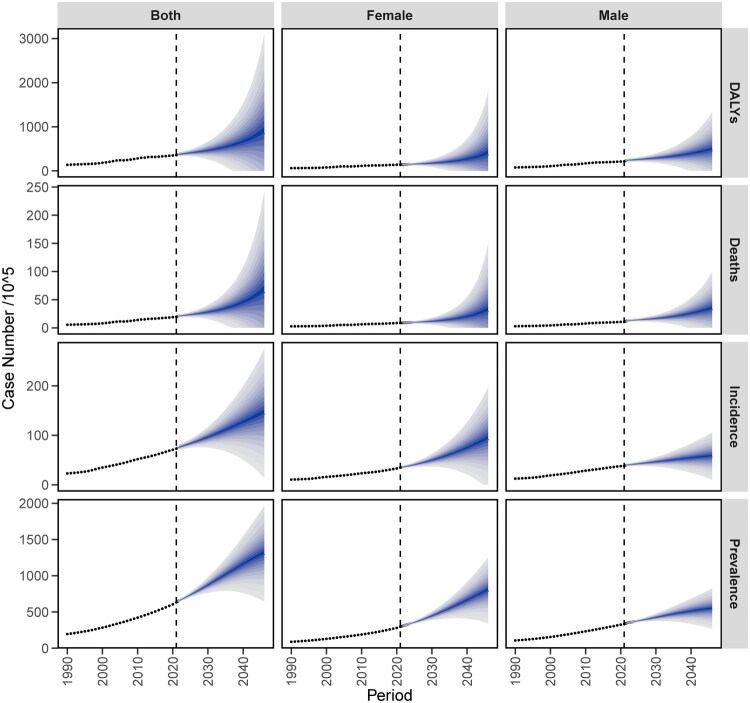
Trends of the number of cases of IHD in China from 2021 to 2046 predicted by the BAPC model from 2021 to 2046.

**Figure 14.**
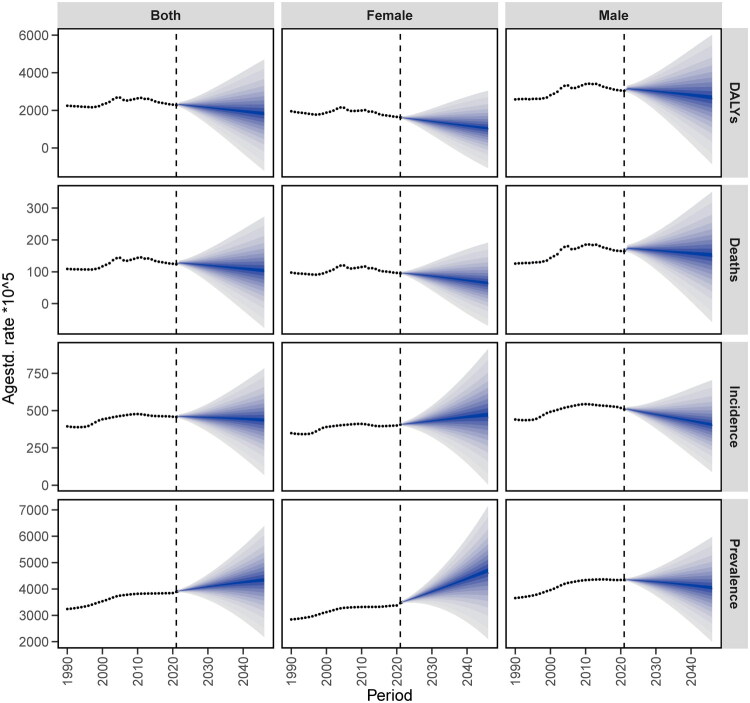
Trend of the standardized rate of IHD in China predicted by the BAPC model from 2021 to 2046.

The analysis of patients with IHD projected to 2046 is illustrated in the figure. As the years progress, the number of people with IHD in China shows a slight increase, reaching an estimated value of 14,410,378 (both) by 2046, with 9,298,986 females and approximately 5,776,586 males. Regarding the ASIR, the total number of IHD patients and males shows a downward trend over the years, reaching approximately 426 and 397 respectively by 2046; meanwhile, the female group shows a slight upward trend, reaching about 459. The number of patients suffering from IHD is projected to slightly increase over the years, with an estimated value of 130,442,713 (both), including approximately 79,840,690 females and about 54,426,826 males. The ASPR for both and females show an upward trend, reaching 4,280 and 4,628 respectively by 2046; the male group remains stable with a slight downward trend, reaching 3,985. The number of deaths among IHD patients shows a slight increase over the years, with an estimated value of 6,391,896 (both) by 2046, including 3,038,431 females and 3,376,280 males. The ASMR has been declining over the years, with totals of 99, 61 for females, and approximately 146 for males. The number of ASDR among IHD patients is projected to slightly increase over the years, with an estimated value of 85,825,567 (both), including approximately 37,688,713 females and about 47,374,647 males. The DALY rate has been declining over the years, with totals of about 1,740, 984 for females, and 2,576.55 for males (Figures 13 and 14).

#### 3.9.2. Nordpred analysis

**Figure 15.**
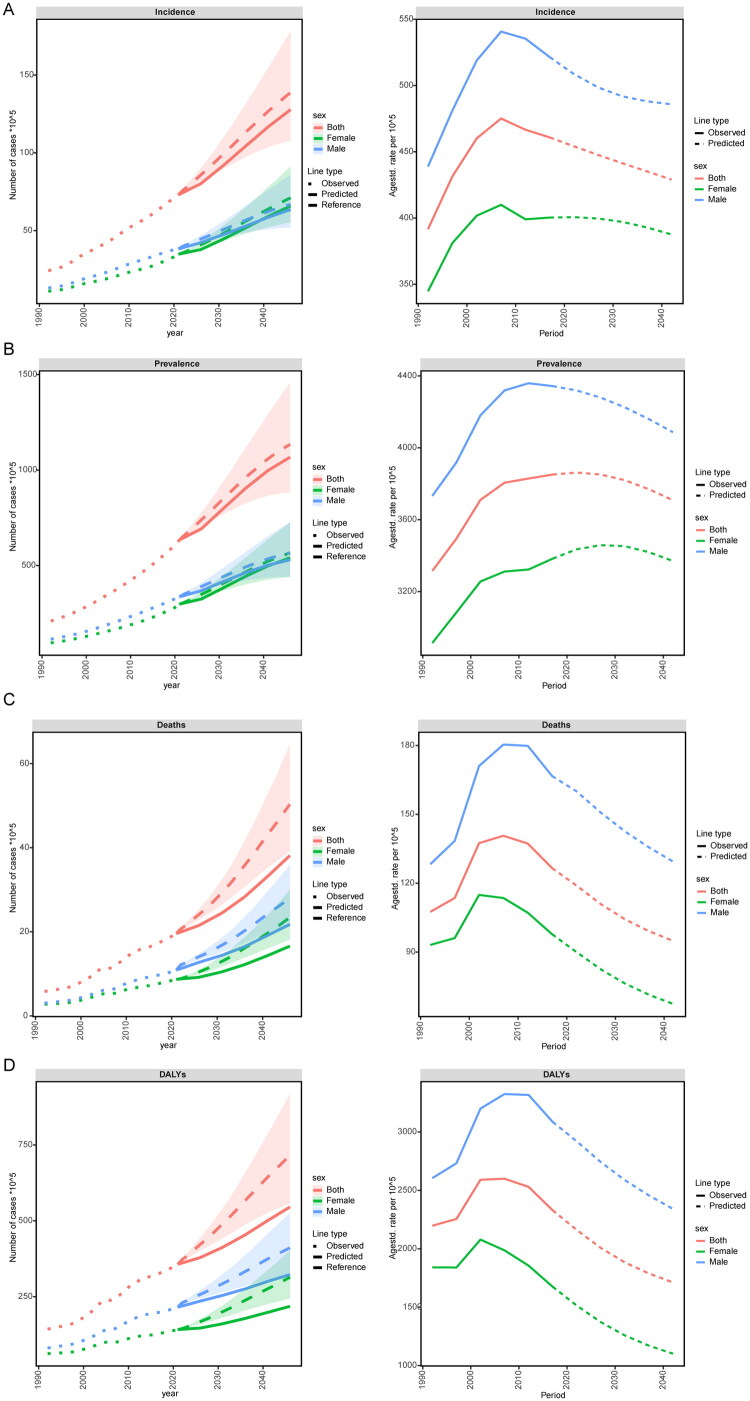
Predictions of the nordpred model for the number of IHD cases and standardized rates in China from 2021 to 2046. (A) Incidence rate; (B) Prevalence rate; (C) Mortality rate; (D) DALYs rate.

The incidence, prevalence, mortality rate, and the number of Disability-Adjusted Life Years (DALYs) due to IHD in China have shown an increasing trend over the years. As shown in the accompanying figure, the number of affected male patients was higher than that of females. By 2021, however, the ASR for incidence, mortality, and DALYs related to IHD in China exhibited a decline. The ASPR for both the overall population and female patients are projected to experience a slight increase before 2030, followed by a downward trend (Figure 15).

#### 3.9.3. Arima analysis

**Figure 16.**
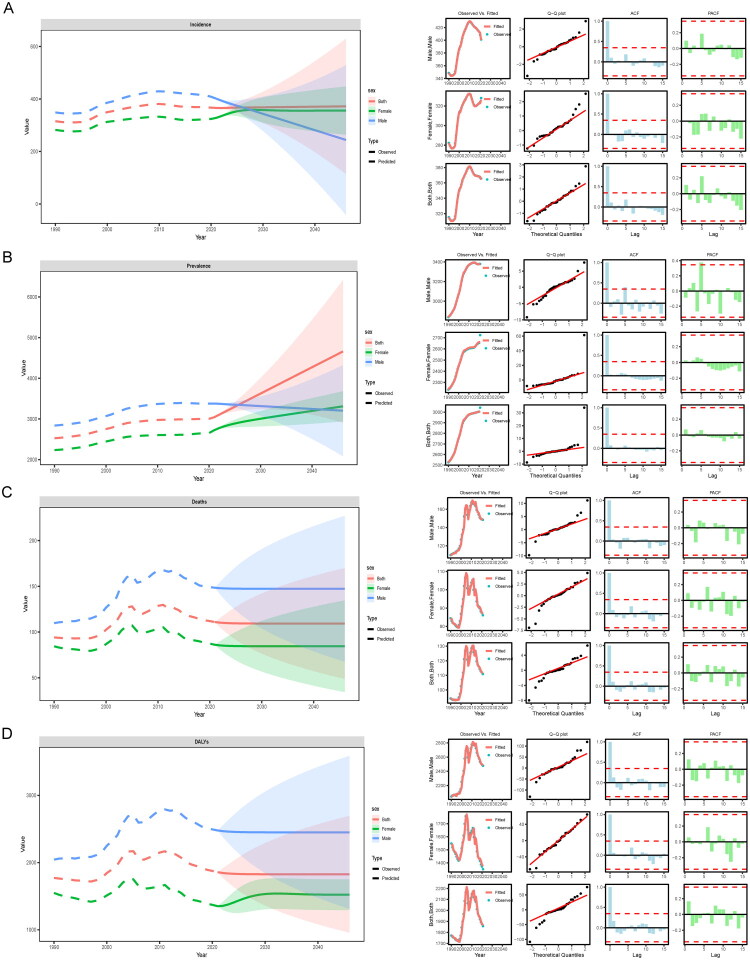
Predictions of the arima model for the trend of standardized rates in China from 2021 to 2046. (A) Incidence rate; (B) Prevalence rate; (C) Mortality rate; (D) DALYs rate.

We employed the ARIMA method to forecast the annual ASR of incidence, prevalence, mortality rate, and DALYs for IHD among male and female populations. All four predictive models met ARIMA-related criteria; further details are presented in the corresponding figures. From 2021 onwards, with each passing year, the predicted values for ASIR among females tend to stabilize after 2021 while those for males show a declining pattern. Regarding ASPR, there is a decrease observed in males while both total population and females exhibit an upward trend. By 2046, it is anticipated that female ASPR will slightly exceed that of males. In terms of ASMR and ASDR, the trends for both genders post-2020 appear to level off. By 2046, predictions indicate that the male values will surpass those of females (Figure 16).

## 4. Discussion

This study conducts a comprehensive analysis based on the latest data regarding the incidence, prevalence, mortality rates, and Disability-Adjusted Life Years (DALYs) of IHD in Belt and Road Initiative countries from 1990 to 2021. The findings indicate a polarized trend in IHD burden among these nations during this period. Some Western European countries, such as Bulgaria, Croatia, Italy, and Portugal, have experienced declines in standardized rates of incidence, prevalence, mortality, and DALYs. About 30% of countries, including China, Russia, the United Arab Emirates, and some Central Asian and Southeast Asian countries, have seen an upward trend in epidemiological indicators. Among them, the growth in China is particularly significant. Furthermore, there are periodic fluctuations in China’s ranking concerning IHD within Belt and Road countries. It is observed that the number of male cases significantly exceeds that of female cases—predominantly affecting individuals aged over 60 with an escalating risk associated with advancing age. The Belt and Road countries include diverse ethnic groups like Arabs, Central Asian Turks, and Han Chinese. Differences in ischemic heart disease (IHD) burden may arise from genetic variations. A meta-analysis found that the T allele and TT genotype of CD14 C-260T polymorphism were significantly linked to IHD risk in East Asians (recessive model OR = 1.53; co-dominant model OR = 1.81), but not in Europeans or Indians [32]. Liu et al. showed that nearly half of East Asians carry the defective ALDH2*2 genotype, highlighting clinical significance for restoring ALDH2 function [33]. In Kazakhs, the T alleles of GCLM gene-588C/T (OR = 3.92) and GCLC gene-129T/C (OR = 3.22) polymorphisms were associated with IHD risk [34]. Among South Indian Tamils, CYP2C19*2/*3 polymorphism reduced clopidogrel response and increased residual platelet activity in IHD patients, suggesting gene-guided personalized antiplatelet therapy [35]. External factors amplify disease risk *via* epigenetic regulation. For instance, a high-salt diet exacerbated hypertension and vascular remodeling in rs1275988C/C carriers, increasing IHD and cardiovascular risks [36]. Hypomethylation of the AHRR gene ROI was linked to obstructive coronary artery disease (CAD) risk (OR = 1.024, *p* = 0.046), serving as a potential biomarker for air pollution-related CAD [37]. Beyond genetics and environment, national medical conditions, social policies, and healthcare efficiency also influence IHD outcomes. In Central Asian countries like Uzbekistan exhibit rising IHD incidence closely linked to economic transformation: industrialization has led to dietary westernization characterized by increased consumption of high-fat high-calorie foods alongside reduced physical activity levels resulting in higher obesity rates; specifically among adults over 20 years old where obesity prevalence has risen by 1%, leading to increases in male (40.2 per 100000 population) and female (38.3 per 100000 population) incidence rates for IHD [38]. Moreover,the lagging development of medical infrastructure within these regions hampers early screening capabilities as well as diagnostic proficiency further exacerbating the spread of IHD. In contrast, countries such as Portugal demonstrate success in controlling IHD due largely to sustained investments in healthcare over time coupled with robust primary care systems. In light of the current challenging circumstances surrounding cardiovascular health issues globally, ‘the Belt and Road Initiative’ presents crucial opportunities for addressing challenges posed by IHD through innovative collaborative efforts.

Subsequently, we conducted a decomposition analysis of countries along the ‘Belt and Road’ initiative, specifically focusing on China, Uzbekistan, and Syria, which ranked high in terms of standardized epidemiological indicators for IHD. The decomposition analysis revealed that the increase in IHD cases in China, Uzbekistan

Syria, Egypt, Iraq and Morocco are mainly driven by population growth and age structure. Population growth and age structure primarily drive demographic changes in Syria, Egypt, Iraq, and Morocco, with age structure playing a more significant role especially in China and Syria. The rise in the elderly population coupled with increased life expectancy due to economic development and improved healthcare has led to an elevated risk of IHD incidence, placing significant pressure on healthcare systems [39,40]. This underscores the necessity for enhanced medical technology exchange and cooperation among these nations as well as the establishment of platforms for sharing health data related to elderly care alongside various preventive measures to address disease trends arising from rapid economic development [41]. In lower Socio-Demographic Index (SDI) countries such as Egypt, Iraq, Morocco, and Uzbekistan, the impact of population growth is particularly pronounced. These nations face challenges including shortages of medical resources, weak public health awareness among citizens, and suboptimal living conditions, all contributing significantly to heightened risks associated with IHD. Additionally, the adoption of Western dietary patterns brought about by globalization exacerbates this disease burden [42,43]. For these Middle Eastern and North African countries, it is imperative to actively collaborate with international organizations to secure assistance in medical resources and technical support while launching large-scale campaigns promoting healthy lifestyles aimed at enhancing public awareness regarding IHD prevention. Countries like Uzbekistan within Central Asia could benefit from regional collaboration by establishing training centers for healthcare professionals, thereby improving service capacity collectively addressing the challenges posed by rising populations concerning IHD control efforts. Southeast Asian nations, such as India with Ayurvedic practices and Thailand with traditional herbal medicine, have unique experiences in cardiovascular prevention. These can be leveraged through partnerships with Chinese traditional medicine institutions for joint research to identify effective components and foster therapeutic innovation [44,45].

The risk factor analysis has revealed some significant and emerging risk factors faced by countries with prominent indicators in the Belt and Road Initiative organization. In China, environmental factors, particularly air pollution, have a significant impact on IHD, underscoring the urgency of promoting synergy between environmental health and public health initiatives. Research indicates that particulate matter can induce the accumulation of monocytes in atherosclerotic plaques, triggering oxidative stress cascades that accelerate early-stage atherosclerosis and promote coronary artery calcification. These processes increase the risk of cardiovascular diseases and are closely associated with mortality and disability rates from IHD among men [46,47]. Hypertension, dietary risks and occupational environmental risk factors are particularly prominent in countries such as Egypt, Uzbekistan and Syria. The latest research shows that the increasing burden of cardiovascular and cerebrovascular diseases in Egypt is mainly due to metabolic syndrome, hypertension, psychosocial stress, and high-fat diets, aligning with our findings [48]. The residents of Uzbekistan mainly consume high-sodium, red meat and processed meat, increasing the risk of ischemic heart disease [38]. Due to long-term economic and social instability, Syrian residents have shifted to high-calorie, high-fat processed foods, while lack of exercise and unhealthy diets have increased the incidence of hypertension and ischemic heart disease [49]. Therefore, adjusting the diet structure may be an effective strategy to reduce hypertension in countries such as Uzbekistan and Syria. Both Morocco and Iraq are threatened by high cholesterol diets. High cholesterol in Moroccan adults is associated with multiple factors such as age, urbanization, health awareness and obesity [50]. Iraq’s traditional diet is dominated by red meat and cheese, and the intake of fruits and vegetables is insufficient, which also leads to the widespread problem in the country.

To tackle cross-regional health challenges, countries must integrate precise policy implementation with coordinated governance. Under the Belt and Road framework, this means implementing targeted interventions based on each country’s needs and building a sustainable public health system through technology and policy coordination. For example, China needs to collaborate with international environmental organizations and research institutions to strengthen the enforcement of environmental regulations. Efforts should be intensified to manage industrial pollution emissions in key control areas such as Beijing-Tianjin-Hebei, promote clean energy usage, and improve air quality [51,52]. The fast-paced lifestyle and intense work pressure associated with urbanization have led to the spread of unhealthy habits among the population, accelerating the progression of IHD. China could leverage platforms like the Belt and Road Initiative to share health management models with participating countries [53], collaboratively develop differentiated health promotion programs, host international forums on healthy lifestyles, and foster cross-national cooperation in healthcare development. Furthermore, workers in low Socio-Demographic Index (SDI) regions predominantly engage in labor-intensive sectors such as mining, construction, and manufacturing. The prevalent exposure to harmful chemicals and noise within these working environments significantly elevates cardiovascular disease risks while potentially exacerbating occupational injury rates [54–56]. Simultaneously, these regions often face challenges related to inadequate medical resources; primary healthcare facilities are typically outdated with shortages of qualified medical personnel compounded by an underdeveloped disease prevention system. To effectively control the rising trend of IHD burden in resource-constrained regions, international policies should prioritize supporting resource-poor countries. Governments should enforce affordable engineering and personal protection programs in high-risk industrial and mining areas to reduce environmental and occupational exposure risks [57]. Health systems should rely on primary care networks and community workers to promote low-cost hypertension screening and ensure the affordability and continuous supply of essential antihypertensive drugs [58]. At the same time, basic examination equipment such as blood pressure monitors and simple electrocardiogram machines should be provided to primary health stations, and personnel should be trained in relevant risk assessment, management, and community monitoring skills. At the national level, cross-departmental collaboration and the utilization of the ‘Belt and Road’ cooperation framework should be carried out to promote special assistance for local technology transfer, capacity building of primary health workers, and optimization of the basic drug supply chain, ensuring policy sustainability. Women residing in high-income or middle-high SDI regions appear more vulnerable to blood pressure-related risks linked to occupational environments, a vulnerability likely stemming from biological differences alongside familial responsibilities combined with professional pressures [59]. Communities are the first line of defense against cardiovascular diseases. The Belt and Road Initiative should enhance health education, carry out screening, maintain gender-specific health records and manage the risks of high-risk women, and establish collaborative networks to effectively prevent ischemic heart disease. In our 2021 analysis concerning risk factors we found chronic kidney dysfunction along with low grain diets emerged prominently among critical determinants contributing towards disability due to IHD. Studies indicate that impaired renal function not only disrupts coagulation systems leading up thrombotic events but its associated inflammatory responses may result endothelial dysfunction alongside accelerated atherosclerosis further heightening probabilities for developing IHD [60,61]. Recently Cepoi et al. comprehensively summarized biomarkers related chronic kidney disease pertinent ischemic heart conditions including angiotensin II, endothelin-1, which corroborate mechanisms linking renal impairment directly correlating IHDs’ pathophysiology [62]. Additionally Shuting et al.’s investigation into mortality rates attributed specifically toward IHD similarly confirmed low grain intake constitutes significant factor influencing prevalence thereof [63]. Future endeavors within the Belt and Road Health Work Plan must prioritize addressing individual dietary behaviors and metabolic vulnerabilities, while emphasizing the impacts of ambient air quality on renal impairments and nutritional aspects in relation to their roles concerning IHD.

The analysis of joinpoint results indicates that the epidemiological trends of IHD in China and five other countries exhibited multiple inflection points during the periods of 2000–2010 and 2010–2020. These inflection points suggest that various factors and certain policy events have influenced the epidemiological trends within different time frames, providing valuable insights for future decision-making regarding IHD trends. Around the 2010, China’s initiation of the National Basic Public Health Service Project in 2009 aimed to offer services including management for chronic diseases such as hypertension and diabetes, which facilitated early detection and intervention for risk factors associated with IHD [64,65]. The observed decline in China’s IHD mortality rate post-2011 further corroborates the effectiveness of a series of implemented measures. Uzbekistan has been continuously advancing its healthcare system reforms by introducing foreign investment projects and enhancing grassroots medical networks, significantly improving cardiovascular disease prevention and treatment levels. Between 2004 and 2010, the World Bank’s ‘Health Project No.2’ provided $40 million in funding support to effectively strengthen screening and diagnostic capabilities for cardiovascular diseases in Uzbekistan [66]. Furthermore, in November 2024, both countries expressed their intention to deepen cooperation on traditional medicine and telemedicine under the framework of a ‘Health Silk Road,’ addressing global public health challenges while serving as a cooperative model for other nations.In contrast, the epidemiological indicators of IHD in Egypt, Iraq, Morocco and Syria have shown an upward trend since 2010. Particularly during the period from 2017 to 2019, the ASIR, ASMR and ASDR related to IHD in Iraq all increased. This trend may be attributed to prolonged social instability leading to severe shortages of healthcare resources coupled with significant attrition among healthcare personnel as well as shortages of essential medical supplies and medications [67,68]. During the period from 2010 to 2015 however these countries witnessed a declining trend in standardized mortality rates due possibly to optimized allocation of medical resources along with stabilization of social conditions combined with reconstruction efforts within public health systems. Over the past nearly 30 years, the ASPR of IHD in these countries has generally shown an upward trend, which may be closely related to the accelerated population growth, intensified aging, and insufficient prevention and control of various complex causes such as metabolic diseases and environmental occupations [69]. This indicates the key points of prevention that need attention in the IHD field in the future. We need to further adopt methods to capture the trend of disease growth in these countries and analyze the weights and causes behind the complex factors in these countries, such as China.

Next, we conducted a Net Drift analysis on the IHD epidemiological data of the Belt and Road countries, and also found that the annual change of IHD among countries was significantly different. The Net Drift indicator of ischaemic heart disease shows that the leading countries in terms of increase in incidence are concentrated in East Asia, Central Asia and Southeast Asia. These regions are located in the core hub areas of the Belt and Road Initiative, where rapid economic development, accelerating urbanization and westernization of lifestyles such as high-fat diets have led to an increase in the prevalence of metabolic syndrome and hypertension, which are important predisposing factors for ischemic diseases. Central Asia, Southeast Asia, West Asia, West Africa and North Africa, which are located in the Belt and Road energy corridor and trade nodes, are affected by occupational exposure (such as miners’ pneumoconiosis) during industrialization, infectious diseases and weak public health systems. For example, air pollution caused by oil export dependence in Niger in West Africa and water quality pollution in Southeast Asian countries both increase the burden of cardiovascular disease [70–72]. Southern and eastern Africa and Oceania, where mortality and DALYs have increased significantly, are located in the key areas of China-Africa cooperation and South Pacific cooperation under the Belt and Road Initiative. These regions face the dual challenges of inadequate medical resources, high poverty rates, and climate change, such as South Africa’s dependence on imported food due to drought, which increases malnutrition related heart disease, and Oceania’s extreme weather, which greatly increases the risk of cardiovascular and cerebrovascular diseases [73,74]. Based on the regional layout of the Belt and Road Initiative and the current situation, the following cooperation can be promoted in different regions: in East Asia, Central Asia and Southeast Asia, strengthen high-risk screening for cardiovascular diseases and the construction of intelligent medical infrastructure; in Central Asia, West Asia, West Africa and North Africa, optimize energy infrastructure and chronic disease management systems, and promote affordable medical technologies. Furthermore, there are significant disparities and deficiencies in healthcare in some regions of the Belt and Road countries. Central Asian countries like Kazakhstan and Tajikistan face shortages of healthcare workers, weak primary care, and overuse of antibiotics. Despite having 70% of the population as medical professionals, many are inadequately trained, leading to high rates of unnecessary hospitalizations (40.5% for children and 69.2% for pregnant women in Tajikistan) [75]. Furthermore, In addition, the electronic medical record coverage is only 36.4%, and 92.5% of hospitalized children receive antibiotics. Basic volunteer programs and other initiatives are needed to address these issues [76]. Southeast Asian countries like Singapore and Thailand are leading in digital and personalized healthcare, but in Cambodia and Laos, the internet penetration rate is less than 70% [77]. Due to the lack of cross-border data sharing and uneven medical resource distribution, the two regions face healthcare inequality worsened by technology costs and the dual burden of infectious and chronic diseases. In Africa and Oceania, we will deepen the construction of climate-resilient medical facilities through the Forum on China-Africa Cooperation, and focus on agricultural nutrition cooperation projects and national climate health warning platforms [78]. Thus, it is essential to strengthen primary healthcare and nursing training, promote standardized treatment, build a regional medical cloud for data sharing, and optimize cross-border resource allocation *via* multilateral cooperation to improve service accessibility.

Further age-cohort-period analysis of IHD in China showed that the population in the middle population is aging seriously, and the risk of IHD is increasing with increasing age, which is consistent with our previous analysis of age characteristics. The 2017–2021 period is higher than any previous period, and judging by the trend of the curve, there is a trend of growth in the next few years. Combined with the results of the previous decomposition analysis, risk factor analysis, and Jionpiont regression analysis, it can be seen that China has a higher burden of IHD than other countries, mainly caused by multiple factors such as changes in dietary structure, metabolic diseases, and air pollution. Economic development has shifted residents’ diets toward a Western pattern, high in fat, protein, sodium, processed meats, and red meat, with insufficient whole grain intake. These are key risk factors for IHD. This unhealthy diet has triggered metabolic diseases such as hypertension, high cholesterol, high blood sugar, and obesity, further exacerbating the burden of IHD [79,80]. Research shows that from 1990 to 2019, the mortality rate and DALYs of IHD caused by metabolic factors in China increased significantly, mainly attributed to the aforementioned risk factors [81]. In recent decades, while China’s rapid economic development has improved residents’ living standards and medical conditions, it has also brought new problems. Studies have shown that unhealthy lifestyles such as lack of exercise, obesity, and sedentary behavior have become emerging risk factors for IHD [82]. At the same time, air pollution significantly impacts health and poses a challenge to economic development. According to one study, the effect of PM2.5 on life distribution was directly estimated by causal modeling, and it was found that if the average annual PM2.5 exposure level was reduced from 12 μg/m³ to 7.5 μg/m³, the life expectancy would increase by about 0.9 years [83]. Young people are more vulnerable to PM2.5, PM10, and NO2 than older adults [84]. Therefore, while paying attention to the elderly, the impact of environmental and complex factors on birth cohorts also needs to be considered.

Based on the results of APC analysis, it is necessary to implement a dual-track prevention and control strategy focusing on precision population and health system. In the basic health care that takes into account conventional indicators and emerging factors such as renal function and whole grain intake, standardized cardiovascular risk assessment should be conducted for elderly patients aged ≥75 years with a high risk of IHD. An IHD multidisciplinary team (MDT) clinic was established for elderly patients with multimorbidity to integrate treatment, nutrition and drug management [85]. For the young cohort born between 2002 and 2006, the government and education departments should implement comprehensive campus health education covering cardiovascular knowledge, whole grain diets, physical exercise, pollution prevention, and traditional Chinese medicine [86,87]. At the same time, maternal nutrition intervention and scientific infant feeding should be strengthened to lay a solid foundation for health. To address the peak effect during 2017–2021, China must expand geriatric cardiovascular specialist resources and enhance high-risk population screening and follow-up *via* the family doctor mechanism. Considering the heterogeneity of IHD risk factors in Belt and Road countries, a standardized transnational risk factor database platform should be established to support collaborative research, precise prevention, and regional governance.

We used three different prediction models to project the evolution data of IHD in China over the next 25 years. BAPC and ARIMA predict that the ASIR for women will remain stable, while that for men will decline slightly, causing the ASIR for women to exceed that for men. BAPC and Nordpred predict an increase in the standardized prevalence rate for women, while it remains stable or declines for men; ARIMA predicts that the prevalence rate for women will surpass that for men by 2046. For standardized mortality rates and DALYs, BAPC and Nordpred show a downward trend, whereas ARIMA predicts stabilization after 2020, with male mortality remaining higher than female mortality. Overall, BAPC and Nordpred exhibit consistent long-term trends, while ARIMA focuses more on gender differences. Model discrepancies may arise from varying sensitivities to cohort effects, time series, and risk factors, requiring further validation using additional databases. In summary, China’s total number of IHD patients is increasing, but age-standardized rates are stable or declining due to advances in medical technology and improved risk factor management. However, the rising incidence and patient numbers are driven by China’s large population base, population aging, the spread of metabolic diseases, and enhanced diagnostic capabilities [88]. Although the current policies are effective, in order to deal with the increasing burden of disease, it is still necessary to further integrate resources and optimize policies to build a complete medical system.

In terms of optimizing medical resources, we recommend establishing a real-time monitoring platform for hospital bed availability based on the Internet of Things (IoT). This platform would integrate data on national hospital bed occupancy rates and CT/MRI equipment utilization through intelligent algorithms to facilitate dynamic allocation of medical resources across regions [89,90]. The construction of disease models could involve collaboration with domestic and international technology companies such as Huawei and DeepSeek to jointly develop spatiotemporal prediction models based on deep learning techniques such as LSTM neural networks [91,92]. By integrating multidimensional information from healthcare data, demographic statistics, environmental pollutant monitoring such as particulate matter and heavy metals, along with health behavior data from social media platforms, we can establish a comprehensive analytical framework that accurately tracks hotspots for IHD risk. Traditional Chinese medicine approaches such as herbal therapy and acupuncture demonstrate unique advantages in the prevention of IHD; thus, they may serve as complementary intervention strategies [93,94]. Building upon this foundation, it is imperative for the government to promote innovation in healthcare service models: on one hand, by developing internet-based healthcare services and telemedicine solutions while constructing cross-border medical network platforms with standardized service protocols. Particularly through remote consultation platforms that break down geographical barriers, patients in remote areas can conveniently access expert resources; on the other hand, expanding online health consultation and monitoring services will enable efficient sharing of high-quality medical resources. we aim to ultimately establish a dynamic response mechanism coupled with multi-level coordination strategies. This approach provides flexible and effective solutions for addressing the evolution of chronic diseases such as IHD.

**Figure 17.**
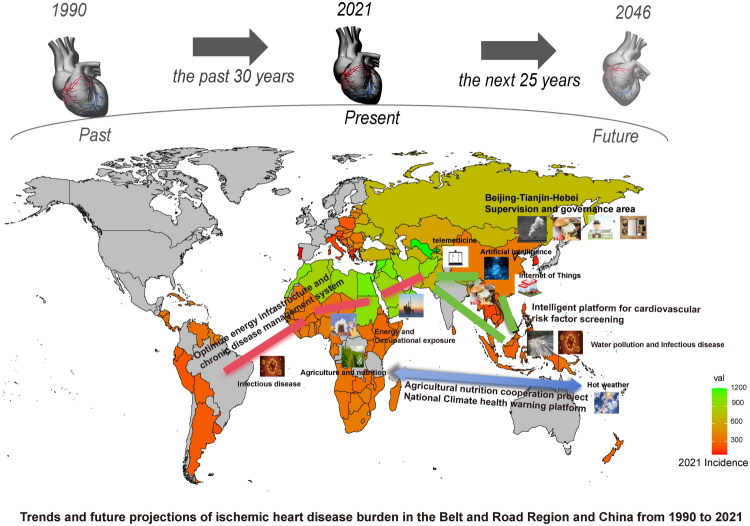
Proposed map of geographical layout based on GBD research in countries along the belt and road and China.

Compared with traditional GBD studies, regional GBD studies have limitations in scope, strategy, and implementation, focusing only on geographic proximity and lacking a substantive framework for cooperation [95,96]. In this study, three breakthroughs were made based on the traditional GBD analysis framework. This is the first time to focus on the Belt and Road Organization, revealing the heterogeneity of disease distribution under the transnational cooperation strategy, and providing an exclusive basis for regional collaborative governance. Based on the analysis of traditional factors such as population growth and aging, three forecasting methods are innovated and integrated, which significantly improves the robustness of forecasting and policy operability. This paper proposes a governance model for the first time under the organizational framework, including cross-border data technology sharing, medical facility transfers, infrastructure projects, and traditional medicine integration. This model provides a clear path for building the Healthy Silk Road (as shown in Figure 17) and offers a solution with both mechanism depth and practical innovation for global cardiovascular disease prevention and control. However, previous literature suggests that GBD studies have some limitations [97]. Although GBD databases incorporate multiple data sources on a global scale, differences in data quality and completeness between different countries and regions may affect the accuracy and reliability of the results. Despite the use of robust statistical methods to reduce bias, some low-income countries, especially those in low-resource areas, still face many challenges in GBD data, such as underreporting, data gaps and shortage of medical resources. These challenges, coupled with inherent limitations such as systematic underreporting and variability in diagnostic/coding criteria, have weakened GBD estimates. This often leads to systematic underestimation of disease burden, especially for diseases dependent on diagnosis, biased distribution across different diseases/populations/regions, and overestimation of broad categories of causes of death due to classification errors. In addition, temporal and geographic differences in reporting and diagnostic practices prevent reliable trend analysis and cross-country comparability.

## 5. Conclusion

This study provides a comprehensive estimation of the overall changes in IHD across Belt and Road countries. From 1990 to 2021, although the standard variation rates of IHD incidence, prevalence, mortality, and disability-adjusted life years (DALYs) fluctuated within a narrow range—with some indicators even showing a decline—the total number of IHD cases and projected annual cases have gradually increased. Furthermore, certain Asian and African countries such as China, Russia, Uzbekistan, Syria, and Egypt continue to bear a significant burden of this disease. The interplay between traditional risk factors such as hypertension and high cholesterol and emerging risks including air pollution, occupational exposure, renal dysfunction, and low grain diets is notably pronounced. In response to the differentiated trends in disease across regional countries, we propose a stratified collaborative governance framework. This framework encompasses three key mechanisms: cross-border data technology sharing, transfer of medical facilities and infrastructure projects, and integration of traditional medicine. These elements together form a closed-loop system, providing practical methods with regional characteristics for the prevention and control of cardiovascular diseases globally.

## Supplementary Material

Supplemental Material

## Data Availability

Publicly available datasets were analyzed in this study. This data can be found at: https://vizhub.healthdata.org/gbd-results/ name of the repository: GBD Results tool.The analytical data can be obtained by contacting the corresponding authors. Tables S1 and S2 can be downloaded from the list below.
